# Perovskite–fullerene hybrid materials suppress hysteresis in planar diodes

**DOI:** 10.1038/ncomms8081

**Published:** 2015-05-08

**Authors:** Jixian Xu, Andrei Buin, Alexander H. Ip, Wei Li, Oleksandr Voznyy, Riccardo Comin, Mingjian Yuan, Seokmin Jeon, Zhijun Ning, Jeffrey J. McDowell, Pongsakorn Kanjanaboos, Jon-Paul Sun, Xinzheng Lan, Li Na Quan, Dong Ha Kim, Ian G. Hill, Peter Maksymovych, Edward H. Sargent

**Affiliations:** 1 Department of Electrical and Computer Engineering, University of Toronto, 10 King's College Road, Toronto, Ontario M5S 3G4, Canada; 2 Center for Nanophase Materials Sciences, Oak Ridge National Laboratory, 1 Bethel Valley Road, Oak Ridge, Tennessee 37831, USA; 3 Department of Physics and Atmospheric Science, Dalhousie University, Room 319, Dunn Building, Halifax, Nova Scotia B3H 4R2, Canada; 4 Department of Chemistry and Nano Science, Ewha Womans University, 52, Ewhayeodae-gil, Seodaemun-gu, Seoul 120-750, Korea

## Abstract

Solution-processed planar perovskite devices are highly desirable in a wide variety of optoelectronic applications; however, they are prone to hysteresis and current instabilities. Here we report the first perovskite–PCBM hybrid solid with significantly reduced hysteresis and recombination loss achieved in a single step. This new material displays an efficient electrically coupled microstructure: PCBM is homogeneously distributed throughout the film at perovskite grain boundaries. The PCBM passivates the key PbI_3_^−^ antisite defects during the perovskite self-assembly, as revealed by theory and experiment. Photoluminescence transient spectroscopy proves that the PCBM phase promotes electron extraction. We showcase this mixed material in planar solar cells that feature low hysteresis and enhanced photovoltage. Using conductive AFM studies, we reveal the memristive properties of perovskite films. We close by positing that PCBM, by tying up both halide-rich antisites and unincorporated halides, reduces electric field-induced anion migration that may give rise to hysteresis and unstable diode behaviour.

## Abstract (web summary)

Metal halide perovskites are promising for solar energy harvesting, but currently prone to a large hysteresis and current instability. Here, Xu *et al*. show improvements in a hybrid material in which the fullerene is distributed at perovskite grain boundaries and thus passivates defects effectively.

## Graphical abstract

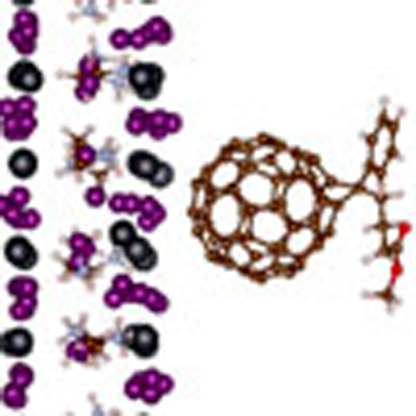

Solution-processed metal halide perovskites, especially the class of methyammonium lead halide perovskites (MAPbX_3_, where MA indicates methylammonium and X the halide), are attractive as solar energy harvesters due to efficient ambipolar transport and strong light absorption. Progress in perovskite photovoltaics has benefited from the use of mesoporous scaffolds^1^,^2^,^3^,^4^ that lessen the need for long minority carrier drift and diffusion by including the active light absorber within nanometre-sized electron-harvesting pores. Effort to improve device performance with such architectures has led to 1,000-h long-term stability 5 and, in separate studies, certified solar power efficiency exceeding 18% (refs 6, 7).

Planar-electrode (as distinct from mesoporous) devices also hold important application such as in photodetector arrays (which demand stringent spatial uniformity pixel to pixel), lasers (which require planarity for low-scatter waveguiding) and flexible photovoltaics (which strive to avoid high-temperature mesoporous oxide processing)^8^,^9^,^10^,^11^,^12^,^13^. The performance of planar perovskite rectifying junction devices has, to date, suffered from two potentially interrelated concerns: severe hysteresis^14^,^15^,^16^, specifically scan-direction- and scan-speed-dependence of photo *J*–*V* characteristics^17^,^18^,^19^; and, relatedly, recombination, likely associated with defective grain boundaries induced by excess halides^20^,^21^,^22^,^23^,^24^. The dependence of hysteresis on device architectures has also been observed, where inverted structures have typically shown less serious hysteresis than regular planar devices, but a lower open-circuit voltage^25^,^26^. There has been, to date, no consensus as to the origins of these findings.

Heterojunction contact engineering has been proposed to address this issue, such as the modification of the TiO_2_ contact layer using a C_60_ self-assembled monolayer^27^. Recent efforts have also sought to reduce hysteresis using interfacial treatment following the formation of perovskite films. However, these solid-state post-treatments typically feature long annealing steps at elevated temperature and therefore introduce undesirable complexity^26^,^28^. Furthermore, addition of passivants to the interface fails to address the defects found throughout the bulk^21^,^29^.

Bearing these considerations in mind, we pursue a solution-phase *in situ* passivation strategy with the goal of enabling simple low-temperature processing and efficient passivation throughout the grain boundaries in the bulk of the perovskite active layer.

## Results

### Improvement of hysteresis and photovoltaic performance

**Figure 1:**
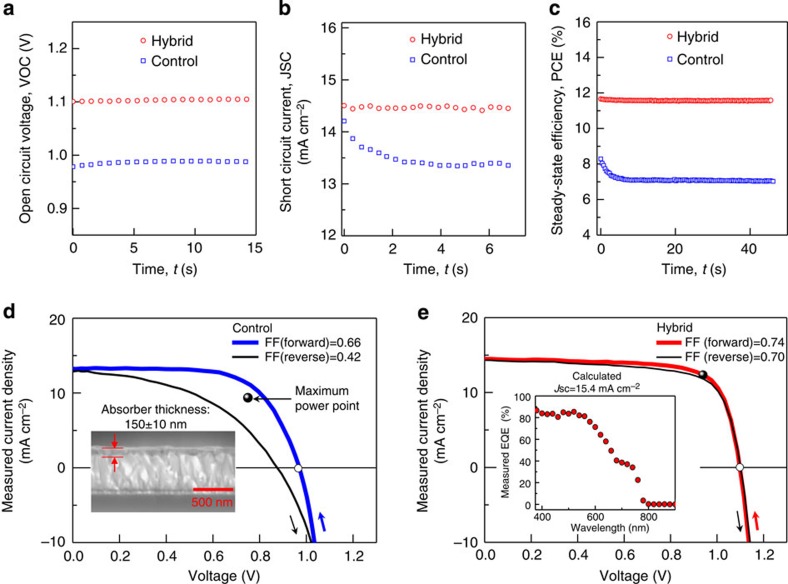
Steady-state photovoltaic performance of an ultrathin perovskite–PCBM hybrid film. (**a**) The steady-state open-circuit voltage, *V*_OC_, (**b**) steady-state short circuit current density, *J*_SC_ and (**c**) the steady-state power conversion efficiency, PCE, of perovskite–PCBM hybrid film (red) compared with the control perovskite-only film (blue). During steady-state measurement, the integrating time for each point is 0.35 s. (**d**) The instantaneous *J*–*V* curve of the control device (perovskite film) with high hysteresis. The thicker curve indicates forward scan starting from open-circuit condition; thin curve is the reverse scan from short circuit condition. The scanning rate is 0.2 V s^−1^. The fill factor (FF) of forward scan is 66% while reverse FF is reduced to 42%. The black point indicates the ‘maximum-power output point (MPP)' is measured from the steady-state PCE as shown in (**c**). The MPP here is located between two instantaneous *J*–*V* curves due to the significant hysteresis and current decay. (**e**) The *J*–*V* scan of a hybrid device shows very low hysteresis and low current loss, as shown in (**b**). The FF for forward (reverse) scan is 74% (70%). The steady-state MPP is consistent with the forward *J*–*V* curve, which demonstrates the stability of the hybrid film. The inset of figure (**e**) shows the external quantum efficiency (EQE) of a hybrid device. The current density predicted from EQE is 15.4 mA cm^−2^, consistent with the steady-state current density measured in (**b**); The inset figure of (**d**) shows the thickness of active layer in both devices is around 150 nm.

In the course of device studies of mixed materials made from solutions containing both perovskites and the electron-acceptor PCBM (phenyl-C_61_-butyric acid methyl ester), we observe an enhancement in photovoltaic performance (Fig. 1a–c) and a reduction in hysteresis (Fig. 1d,e) relative to control devices based on perovskites alone, and also compared with separate-layer PCBM–perovskite devices (Table 1, see Supplementary Figs 1 and 2 for different device structures). To create the mixed-material films, we disperse PCBM and lead acetate (Pb(Ac)_2_; ref. 20) in various ratios and form films using a one-step process^3^,^9^,^30^,^31^ employing methylammonium iodide (MAI) as the organohalide precursor (see Methods). As well as observing reduced hysteresis (Fig. 1d,e), we observe in the perovskite–PCBM mixed-material device a substantial voltage enhancement (∼0.1 V; Fig. 1a) and a higher fill factor compared with the PCBM-free and bilayer PCBM–perovskite controls (Table 1).

### Mechanistic study of perovkite–PCBM interaction

We proceed to seek mechanistic insights regarding the role, or roles, of the PCBM. Specifically, we asked whether the PCBM could interact with certain chemical species in the mixed-material solution and whether studies of incorporation into films using the new process indicated a homogeneous distribution of PCBM throughout the active layer, compared with segregation into a bilayer device with PCBM either substantially below or above the perovskite.

**Figure 2:**
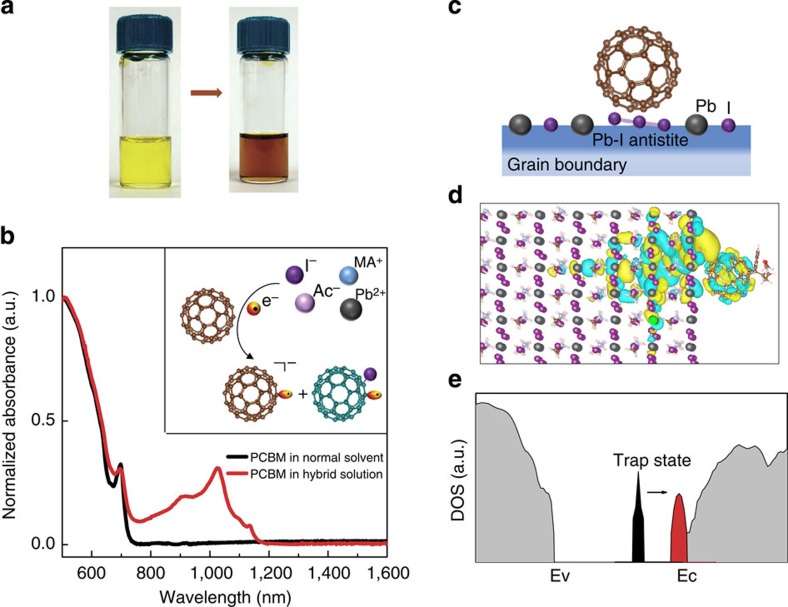
Perovskite–PCBM hybrid process and *in situ* passivation mechanism. (**a**) Pristine perovskite solution (left) comprised of Pb(Ac)_2_ and MAI in dimethylformamide solvent is bright yellow; the formulated perovskite–PCBM hybrid solution (right) is brown; Simple one-step spin-coating is used to convert the hybrid solution into an hybrid solid film, and the perovskite is *in situ* passivated by PCBM during self-assembly; (**b**) Ultraviolet–visible absorption spectroscopy of the hybrid solution shows the interaction between PCBM and perovksite ions. PCBM radical anion's absorption peak at 1,020 nm is identified in hybrid solution (red); while PCBM in same solvent (black) has no absorption peak in this wavelength region; Inset of (**b**) shows details of such interaction: In hybrid solution, electron transfer is induced between the perovskite anions (I^−^) and PCBM and will result in PCBM radical anion and PCBM–halide radical. (**c**) A schematic of *in situ* passivation of halide-induced deep trap: PCBM adsorbs on Pb-I antistite defective grain boundary during perovskite self-assembly. (**d**) The wavefunction overlap shows the hybridization between PCBM and defective surface, enabling the electron/hole transfer for absorbance and passivation. (**e**) DFT calculation of density of states (DOS) shows that deep trap state (black) induced by Pb-I antistite defect is reduced and becomes much shallower (red) upon the adsorption of PCBM on defective halide. Ec, the minimum of conduction band; Ev, maximum of valence band.

Solution-phase spectroscopy provides one means to study the formation of complexes of PCBM with the various perovskite solution-phase precursors. When PCBM is mixed into our normal perovskite precursor solution, the bright yellow solution (Fig. 2a, left) turns dark brown (Fig. 2a, right). The absorption spectrum of perovskite–PCBM hybrid solution shows a peak at 1,020 nm (Fig. 2b)^32^,^33^,^34^. This is in contrast with pure PCBM in the same solvent, which is observed to be transparent in this wavelength region. The 1,020 nm spectral feature is associated in literature reports with the formation of a PCBM–halide radical (Fig. 2b, inset)^32^,^33^,^34^.

This reconfirmation of the strong PCBM–iodide interaction motivates us to explore, using density functional theory (DFT, see Supplementary Note 1 for details), what might occur in a solid material. We look in particular at reactions PCBM might participate in at the excess-halide-associated defects at grain boundaries previously reported to be a dominant source of electronic traps in lead MAI perovskites^20^,^21^,^22^,^23^. We focus specifically on the Pb-I antisite defect, in which iodine occupies the Pb site and forms a trimer with neighbouring iodine atoms (Fig. 2c, Supplementary Fig. 7)^20^. DFT reveals that, with the introduction of PCBM near such Pb-I antisite defects, the wavefunction of the ground state (Fig. 2d) is hybridized between the PCBM and the perovskite surface. We also find that the bonding of PCBM to defective halides is thermodynamically favoured (see binding energy calculations in Supplementary Figs 3–5 and Supplementary Table 1–3) and that this suppresses the formation of deep traps (Fig. 2e, Supplementary Fig. 6).

### Perovskite–PCBM mixture phase distribution

**Figure 3:**
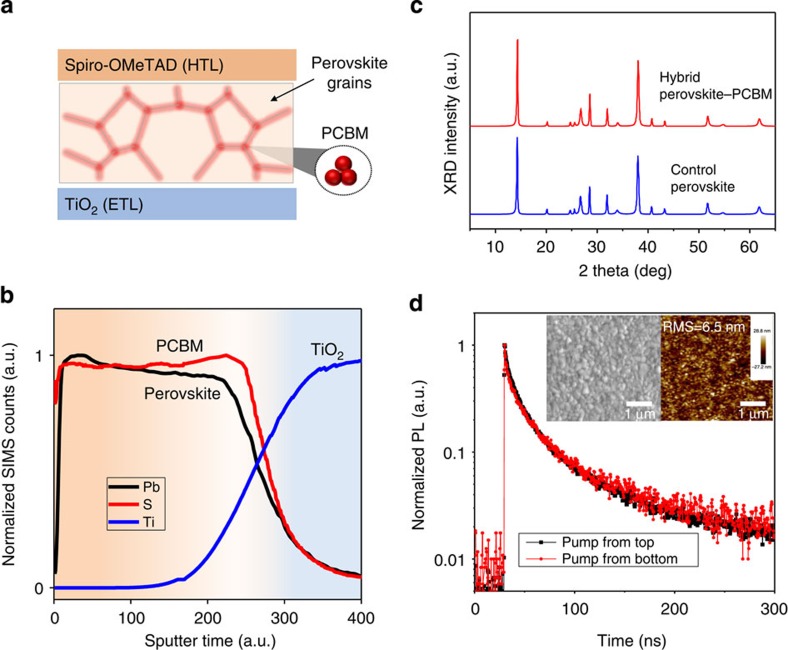
Three-dimensional phase separation and homogeneous PCBM distribution in hybrid solid. (**a**) Scheme of planar perovskite solar cell using perovskite–PCBM hybrid solid as the active absorber; PCBM phase is homogeneously distributed at grain boundaries throughout the perovskite layer. (**b**) SIMS depth profile of perovskite–PCBM hybrid film on TiO_2_ substrate showing homogeneous distribution of PCBM throughout the film. The sputter etching begins at the air/film interface. PCBM is tracked by S element using analogous [60]ThPCBM; perovskite is tracked by Pb element; TiO_2_ is tracked by Ti atom. (**c**) XRD pattern of pristine hybrid solid film (red) and the control perovskite film without PCBM (black). TiO_2_ compact layer on FTO is used as substrate. XRD shows that in hybrid solid, the perovskite crystal lattice is same as control film, and thus PCBM only exists at the grain boundaries and interfaces throughout the film. (**d**) The transient photoluminescence of the hybrid film. Pumping from top of film (black) and pumping from bottom of film (red) give identical signals, showing PCBM homogeneous distribution. The hybrid film is displays dense grains and full-coverage as observed via SEM (inset left); The surface is ultra-flat with roughness ∼6 nm as characterized by AFM (inset right).

Next we seek to determine whether the PCBM is distributed throughout the entire thickness of the active layer that had been formed from the mixed perovskite–PCBM solution (Fig. 3a). Secondary ion mass spectrometry (SIMS) is used to probe the depth profile of PCBM and perovskite. Pb and Ti are used as indicators of the perovskite and of the TiO_2_ substrate, respectively. Since PCBM does not contain elements to identify it uniquely, we use instead for this portion of the study a thiophene-containing derivative, [60]ThCBM ([6,6]-(2-Thienyl)-C_61_-butyric acid methyl ester), which permits the use of sulfur as the tracer element^35^. The [60]ThCBM is present homogeneously throughout the thickness of hybrid film, with a uniform concentration as a function of depth (Fig. 3b, Supplementary Figs 18 and 19, Supplementary Note 2). Using X-ray diffraction (XRD), we find that the perovskite lattice diffraction peaks of the hybrid film are consistent with that of control film without PCBM (Fig. 3c). In addition, the average perovskite grain size in hybrid films, estimated from the XRD peak width, is comparable to that of control film (Supplementary Table 4, Supplementary Methods).

Also with the nature of the mixed material in mind, we employ Kelvin probe studies to examine the work function of mixed-material films. The work function of the mixed-material films lies between that of the pure perovskite and pure PCBM. Its value varies monotonically along this continuum as a function of PCBM fraction incorporated (Supplementary Fig. 10). When very high PCBM fractions are employed, evidence of phase separation and impact on film morphology emerge: the PCBM phase aggregates at perovskite grain boundaries and becomes clearly evident (Supplementary Figs 11 and 12).

These findings prompt us to posit the following picture of the mixed material. Perovskite grains are formed with similar size and crystallinity with and without the PCBM (XRD). The PCBM is distributed uniformly throughout the thickness of the film (SIMS), presumably in between the grains. The PCBM could bind iodide-rich defect sites on these grain boundaries (DFT) and/or simply bind up excess iodide from the solution. From an electronic standpoint, the incorporation of the PCBM throughout the film influences its work function in proportion with PCBM–perovskite ratio (Kelvin probe).

### Charge dynamics and hysteresis characterization

To seek further indications regarding the extent of electronic interaction between the PCBM and the perovskite grains in the mixed material, we acquire transient photoluminescence for pure perovskites, PCBM–perovskite bilayers and mixed materials, investigating each for the case of excitation from each side. The mixed-material film shows identical transient PL (photoluminescence) traces for top versus bottom illumination (Fig. 3d). In contrast, perovskite films with PCBM on one side exhibit different PL lifetimes when pumped from the different sides (Supplementary Fig. 14). The invariance of the PL lifetime with top-/bottom-side photoexcitation for the mixed-material system agrees with SIMS and further indicates that the hybrid film behaves as a homogenous optoelectronic material throughout its thickness.

**Figure 4:**
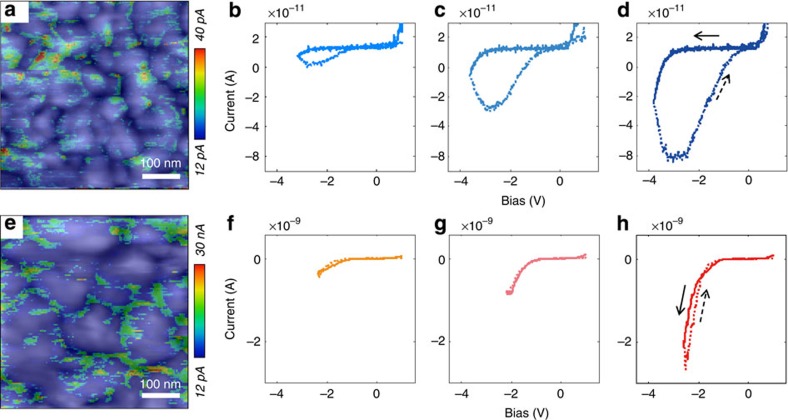
cAFM study of hysteresis-ion relationship for control films (**a**,**b**,**c**,**d**) and hybrid films (**e**,**f**,**g**,**h**). (**a**,**e**) The grey-scaled contact-mode AFM (background) with overlaid colour-scaled conductive AFM images (positive bias voltage: 1 V). (**b**–**d**) *I*–*V* hysteresis of control film increases when increase the negative bias and injected current (solid line, forward sweep; dashed line, reverse sweep). (**f**–**h**) *I*–*V* hysteresis of hybrid film is suppressed when increasing the negative bias and injected current. Scanning rate is ∼0.5 V s^−1^ (see Methods).

A series of conductive atomic force microscopy (cAFM) studies provides added spatial resolution of the electronic properties of the films under study. We carry out the cAFM studies under high vacuum and dark conditions to rule out the effect of light and moisture. By overlapping the grain topography and the electrical current map of the films (Fig. 4a,e), we find that conductivity is greatest at grain boundaries, both in the pure-perovskite and in the mixed-material films. However, the mixed-material films have much higher conductivity near grain boundaries at positive bias voltages, consistent with the electron-transport medium PCBM accumulating near grain boundaries and providing continuous pathways for electron egress. We also obtain *I*–*V* traces at various spatial positions and find that control perovskite films exhibit major hysteresis behaviour when scanned in the reverse bias direction (Fig. 4b–d). Given the pure perovskites' slow response on the seconds timescale, the hysteretic *I*–*V* curves are consistent with the proposed hysteresis mechanism of ionic transport in perovskite solids^36^,^37^,^38^,^39^. To our knowledge, this is the first direct experimental observation of memristive properties within the perovskite material itself via cAFM^39^. This observation is generally in agreement with the very recently reported ionic motion processes in CH_3_NH_3_PbI_3_ perovskite materials^25^,^40^. In contrast, in perovskite–PCBM mixed film, the hysteresis effect is greatly suppressed under all conditions (Fig. 4f–h, Supplementary Fig. 17). These observations further substantiate a picture in which PCBM influences electronic properties when it associates with the perovskite grains at their grain boundaries.

Additional device studies offer further information about the role of PCBM in perovskite device performance and hysteresis. Planar devices incorporating PCBM—whether at an interface or throughout in bulk—are consistently superior in performance to control devices without PCBM (Table 1, Supplementary Figs 13 and 15). Incorporating the PCBM into the film becomes even more advantageous to collect current for thicker active layers, suggesting that the PCBM accepts photocharges and assists in their extraction to the TiO_2_. The champion planar devices were obtained using the perovskite–PCBM mixed material and exhibited steady-state power conversion efficiency (PCE) exceeding 14.4% (Supplementary Fig. 13), 1.5 times more efficient than our PCBM-free perovskite controls.

We also investigate the reverse saturation current density in the various devices and found that the hybrid films consistently reduced the dark current by two orders of magnitude. Rectifying behaviour is also maintained much longer in the mixed material compared with perovskite controls (Supplementary Fig. 16).

**Figure 5:**
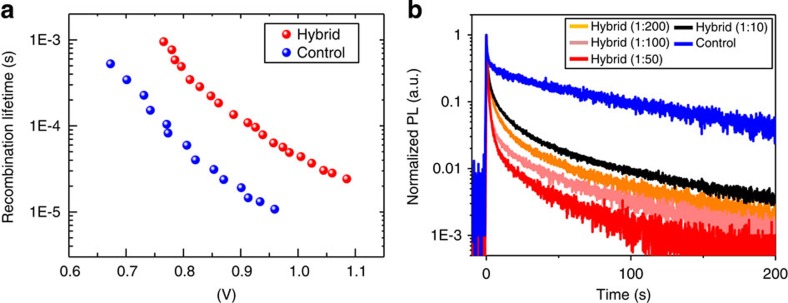
Effect of PCBM on charge carrier dynamics. (**a**) Charge carrier lifetime of hybrid device (red) and control device (blue), determined from transient photovoltage measurement under open-circuit condition. (**b**) Transient photoluminescence of hybrid films with increasing PCBM ratio progressively (orange, pink, red and black) compared with control film on glass (blue), showing the enhanced electron extraction. The quenching efficiency increases monotonically with increasing hybrid ratio, indicating the increasing PCBM–perovskite interfaces. When keep increasing PCBM hybrid ratio (black), the quenching efficiency begins to reduce abnormally, due to the emergence of large domains of agglomerated PCBM (Supplementary Figs 11 and 12), reducing the effective interconnectivity between perovskite and PCBM.

These last observations motivate further evaluation of role of PCBM in trap passivation at perovskite grain boundaries. We use transient photovoltage to quantify the prevalence of mid-gap trap states in each class of materials and devices (details see Methods). We obtain a notably longer carrier lifetime over a wide range of photovoltages in the mixed material (Fig. 5a). This indicates reduced non-geminate recombination for the perovskite–PCBM hybrid films. We also compare the transient photoluminescence of hybrid films to investigate the impact of PCBM on carrier extraction. When we increase the PCBM–perovskite hybrid ratio progressively, the PL exhibits consistently greater quenching, indicating efficient electronic coupling between the well-dispersed PCBM phase and the perovskite (Fig. 5b, orange, pink and red curve). When PCBM ratio is extremely high (Fig. 5b, black curve), the photoluminescence quenching efficiency began to degrade greatly. Significant phase segregation occurs, with the appearance of large PCBM domains that lack effective interconnectivity for carrier extraction (Supplementary Figs 11 and 12). We conclude that comprehensive incorporation of PCBM in the interstitial volumes among grains in the perovskite system is required (that is, sufficient PCBM material miscibility in the perovskite solid is needed) to produce continuous pathways for carrier extraction to enhance performance^41^.

## Discussion

We close with a discussion of mechanisms likely at work, and one more speculative mechanism, in the mixed-material films. Our data suggest that PCBM, when incorporated at or near perovskite grain boundaries, makes a significant impact on electronic properties. The transient photovoltage, combined with the DFT analysis and the spectroscopy showing PCBM radical formation, suggest that PCBM plays a passivating role at iodide-rich trap sites on the surfaces of these grains. At the same time, the long timescale of hysteresis in pure-perovskite films and its substantial suppression in the mixed material, combined with the vastly lower reverse dark current in the mixed material, suggest to us an additional effect at work in addition to the passivating role. We propose that ions, such as the iodide anion, can potentially migrate under an applied electric field, producing an ionic current. This can explain the slow response of hysteresis^36^,^39^ and the instability of the dark current when pure-perovskite and bilayer devices are employed. By tying up iodide-rich surface sites, or simply unincorporated iodide anions, PCBM can reduce anion migration through defects at grain boundaries^36^,^37^. This rearrangement under external, and also built-in internal, electric fields, could account for solar cell hysteresis. For example, when the device is poised at the *J*_SC_ condition, the large built-in field may induce anionic charge motion that works against this field, leading to a drop in photocurrent in time. A relatively rapid scan towards *V*_OC_ will therefore suffer from low photocurrents; whereas, following an extended pause at *V*_OC_, during which anions can diffuse back to equilibrium positions, a rapid scan to *J*_SC_ will feature a high current in view of the lack of charge compensating the built-in field.

## Methods

### Perovskite–PCBM hybrid solution preparation

Lead(II) acetate trihydrate (Sigma-Aldrich, 99.99%) is dehydrated before use. Then, the dehydrated lead acetate (Pb(Ac)_2_) and MAI (Dyesol, 99%+) are dissolved in dimethylformamide (*N,N*-dimethylformamide, Sigma-Aldrich, 99.9%) with the molar ratio 1:3 to form the perovskite precursor solution. To obtain ultrathin films and thick films, we tune the perovskite concentration between 0.2 and 1 mM. For hybrid solid, PCBM (Nano-C, 99.5%) is mixed into the perovskite solution. In typical procedure, the PCBM-perovskite weight ratios are between 1:100 and 1:10. Specifically, PCBM can be dissolved into chlorobenzene first, and then mixed with perovskite solution before spin-coating. The solution is kept at 70 °C before spinning. For low mixture ratio, the miscibility of mixture solution is good and can be stabilized at room temperature; for high-ratio mixtures approaching 1:10 and beyond, the solution needs to be used quickly after mixing.

### Planar solar cell fabrication

A thin TiO_2_ compact layer is first formed on fluorine doped tin oxide (FTO) substrate using magnetron sputtering (∼50 nm, Kurt J. Lesker, 99.9%) followed by a low-concentration TiCl_4_ treatment for interfacial contact improvement^4^,^42^: soak in 120 mM TiCl_4_ aqueous solution at 70 °C for 0.5 h followed by annealing at 500 °C for 0.5 h. The TiCl_4_ treatment modifies the roughness without changing the planar structure of TiO_2_ compact layer and final device (Supplementary Fig. 2). Perovskite–PCBM hybrid solid films are deposited on pre-heated TiO_2_ substrate using spin-coating at 3,000–5,000 r.p.m. for 60 s in a nitrogen glovebox. During the spin-coating, the film turns to dark brown, implying that the perovskite crystallization is almost done. The hybrid solid film is then heated for 10 min at 70 °C to remove the residual solvent. For a control planar heterojunction device, pure-perovskite solution is deposited on TiO_2_ substrate in the same way. No excess acetate (Ac^−^) and methylammonium (MA^−^) are found in the final films (Supplementary Figs 8 and 9, Supplementary Methods). For bilayer control devices, PCBM in chlorobenzene (∼20 mg ml^−1^) is spin cast on a TiCl_4_-treated TiO_2_ substrate and then annealed at 70 °C for 10 min before spin-coating the perovskite on top. A thin PCBM layer (<30 nm) between TiO_2_ and perovskite is formed (Supplementary Fig. 20). Hole transfer layer is deposited by spin-coating of Spiro-OMeTAD (Borun Chemical, 99%+) solution following the doping procedure reported in literature^1^. Top contact is 50 nm thermally evaporated gold through the shadow mask under 10^−7^ torr vacuum using an Angstrom Engineering deposition system.

### Steady-state power conversion efficiency characterization

Steady-state open-circuit voltage, *V*_OC_(*t*), is first measured by Keithley 2400 by fixing the current to zero and sampling the voltage at multiple time points. Steady-state short-circuit current, *J*_SC_(*t*), is measured by Keithley while setting the bias voltage to zero and sampling the current at multiple time points. Instantaneous *J*–*V* curves are then measured with a scanning rate of 0.2 V s^−1^, and the voltage of maximum power point (*V*_MPP_) is determined from the instantaneous *J*–*V* curve. Steady-state PCE, PCE(*t*), is measured by setting the bias voltage to the estimated *V*_MPP_. Under the bias of *V*_MPP_, current density value are sampled during a long time period to get *J*_MPP_(*t*). The PCE(*t*) is obtained by the multiplication of *V*_MPP_ and *J*_MPP_(*t*). The active area is determined by the aperture before the solar cell to avoid overestimating the photocurrent. Through this aperture (area 0.049 cm^2^), the illumination intensity was calibrated using a Melles–Griot broadband power meter and set to be 1 sun (100 mW cm^−2^). The AM1.5 solar power is supplied by a class A (<25% spectral mismatch) solar simulator (ScienceTech). The spectral mismatch of the system was characterized using a calibrated reference solar cell (Newport). The total accuracy of the AM1.5 power conversion efficiency measurements was estimated to be ±5%.

### Conductive atomic force microscope characterization

Scanning probe microscopy experiments are carried out in a commercial ultrahigh-vacuum atomic force microscope (UHV bean-deflection AFM, Omicron) using Cr/Pt-coated silicon cantilevers (Budget Sensor, Multi75E-G). All the measurements are performed at a background pressure of <2 × 10^−10^ Torr after transferring the samples from ambient without any additional treatment. Contact-mode AFM images and two-dimensional current maps are simultaneously obtained with the tip in contact with the surface (loading force ∼1 nN) applying fixed bias voltages. The *I*–*V* curves are acquired in the conductive AFM regime from various locations of the sample surfaces applying a linear bias ramp with a rate of ∼0.5 V s^−1^.

### Other characterizations

External quantum efficiency spectrum and transient photovoltage measurements are carried out following previously published processes^42^. Transient photoluminescence is carried out using time-correlated single photon counting (TCSPC) function of a HORIBA Fluorolog-3 spectrofluorometer, and following the method shown in literature to protect samples^43^. Samples are tested in N_2_ ambient.

## Author contributions

J.X., A.B. and E.H.S. designed and directed this study, analysed results and co-wrote the manuscript; J.X. and W.L contributed to all experimental work; A.B. carried out the DFT simulations; O.V. and A.H.I. assisted on experiment design, results analysis and manuscript preparation; R.C. carried out electronic property characterization and transient photoluminescence; M.Y., Z.N., X.L., L.Q. and D.H.K. assisted the device fabrication and characterization; S.J. and P.M. carried out cAFM studies; J.P.S. carried out Kelvin probe study; P.K. and J.M. assisted in microscopic studies.

## Additional information

**How to cite this article:** Xu, J. *et al*. Perovskite–fullerene hybrid materials suppress hysteresis in planar diodes. *Nat. Commun*. 6:7081 doi: 10.1038/ncomms8081 (2015).

## Supplementary Material

Supplementary Information Supplementary Figures 1-20, Supplementary Tables 1-4, Supplementary Notes 1-2, Supplementary Methods and Supplementary References

## Figures and Tables

**Table 1:** Statistics of steady-state performance with different PCBM distribution and thickness.

**Device configuration**		**Steady-state performance**			**Instantaneous**
**Type**	**Thickness (nm)**	***V***_**OC**_ **(V)**	***J***_**SC**_ **(mA cm**^**−2**^)	**PCE (%)**	**FF (%) forward/reverse**
Control	150±20	0.97±0.02	13.1±0.8	6.7±0.5	62/38±3
Bilayer		1.08±0.02	14.2±0.4	10.6±0.4	71/64±3
Hybrid		1.09±0.02	14.4±0.4	10.9±0.4	72/65±2
Hybrid champion		1.11	14.6	11.9	73/68
Control	300±20	0.98±0.02	14.4±0.8	8.1±0.5	65/40±3
Bilayer		1.06±0.02	16.1±0.4	12.0±0.5	72/56±3
Hybrid		1.07±0.02	17.3±0.4	13.6±0.6	73/66±3
Hybrid champion		1.086	18.0	14.4	75/69

FF, fill factor.
