# Supplementary material for: Perovskite–fullerene hybrid materials suppress hysteresis in planar diodes
Source: Nat Commun. 2015 May 8;6:7081. doi: 10.1038/ncomms8081 (PMC4432582; doi:10.1038/ncomms8081)
Supplement: Supplementary Information — Supplementary Figures 1-20, Supplementary Tables 1-4, Supplementary Notes 1-2, Supplementary Methods and Supplementary References [file ncomms8081-s1.pdf]

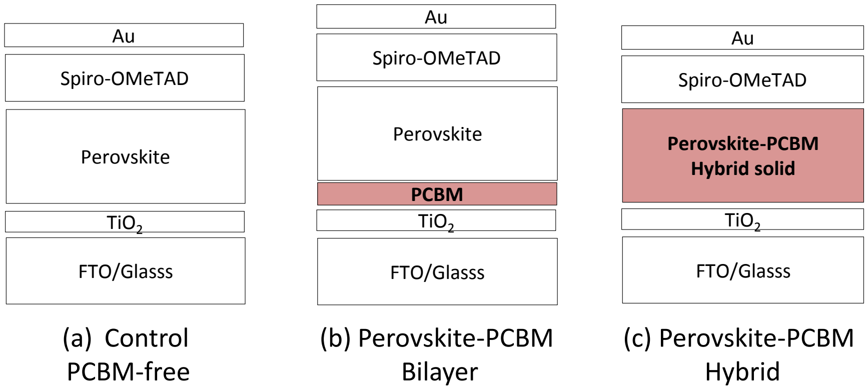

Supplementary Figure 1 | Solution-processed planar device structures in this study. (a) Control device with PCBM-free pure perovskite ($CH_3NH_3PbI_3$) as active layer; TiO_2 and Spiro-OMeTAD as electron transport layer (ETL) and hole transport layer (HTL), respectively.**(b)** Perovskite-PCBM bilayer structure with PCBM cast on TiO_2 before perovskite deposition; and **(c)** Perovskite-PCBM hybrid device with mixed material as active layer.

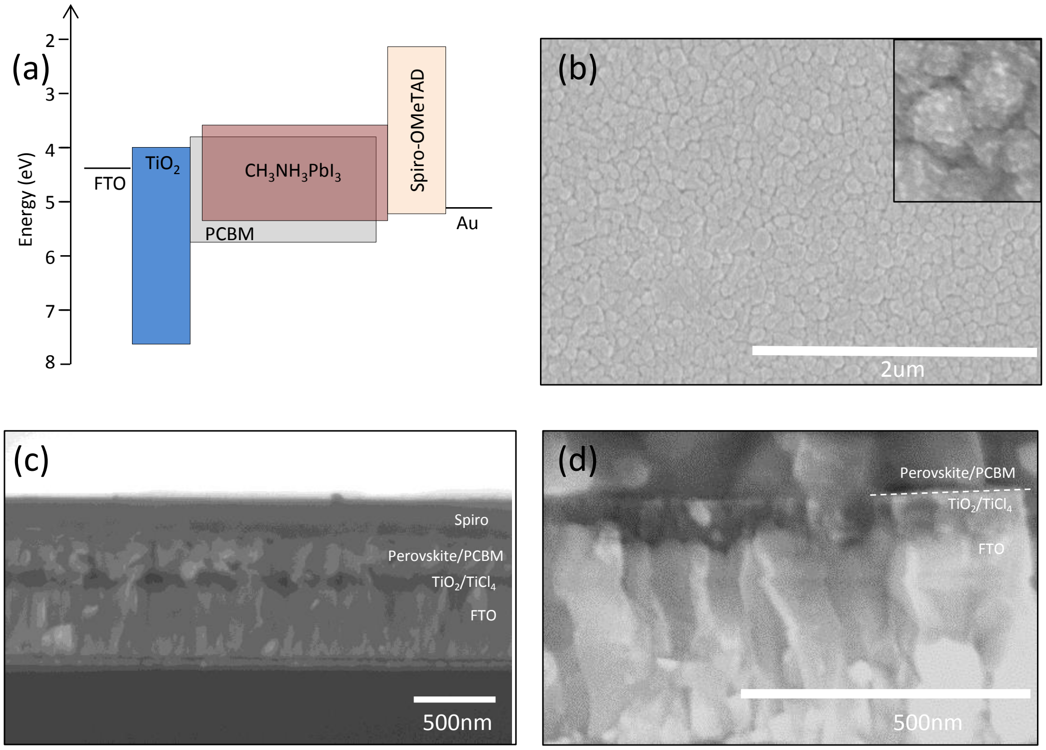

Supplementary Figure 2 | Microscopic study of planar perovskite cell structure. (a) Energy diagram of planar device; **(b)** Top surface morphology of TiO_2 planar compact layer after $TiCl_4$ treatment; and (**inset of b**) zoom-in to show the $TiCl_4$ interfacial modification effect on TiO_2 compact layer; **(c)** Cross-section of a planar perovskite cell; **(d)** Zoom-in of the planar interface between TiO_2 and perovskite active layer.

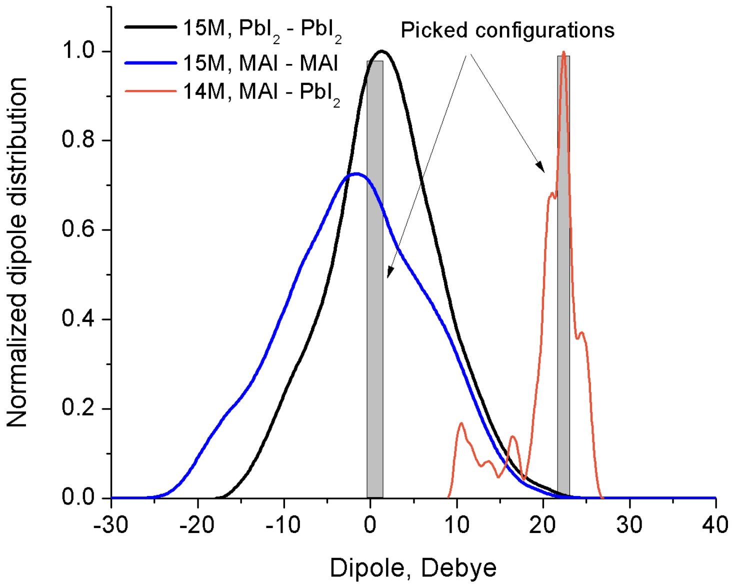

Supplementary Figure 3 | Normalized dipole distribution of symmetrical slabs over the 10 ps period. Shaded area indicates the region from which configurations were selected to be used in passivation studies after appropriate geometry optimization.

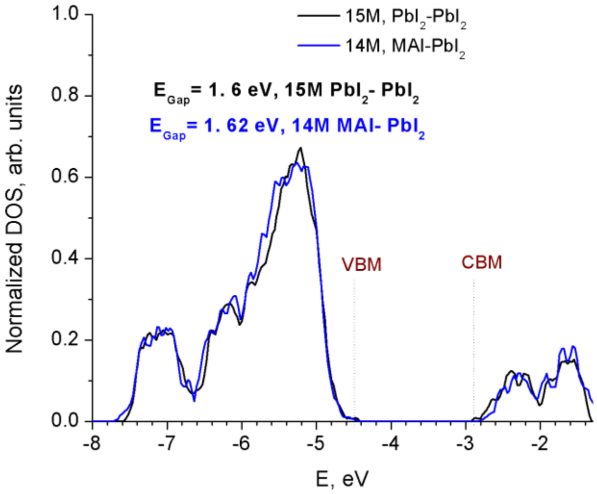

Supplementary Figure 4 | Normalized DOS of the symmetric (PbI_2 - PbI_2) and assymetric (MAI-PbI_2) slabs. The two plots have been aligned using deep lying Pb-levels.

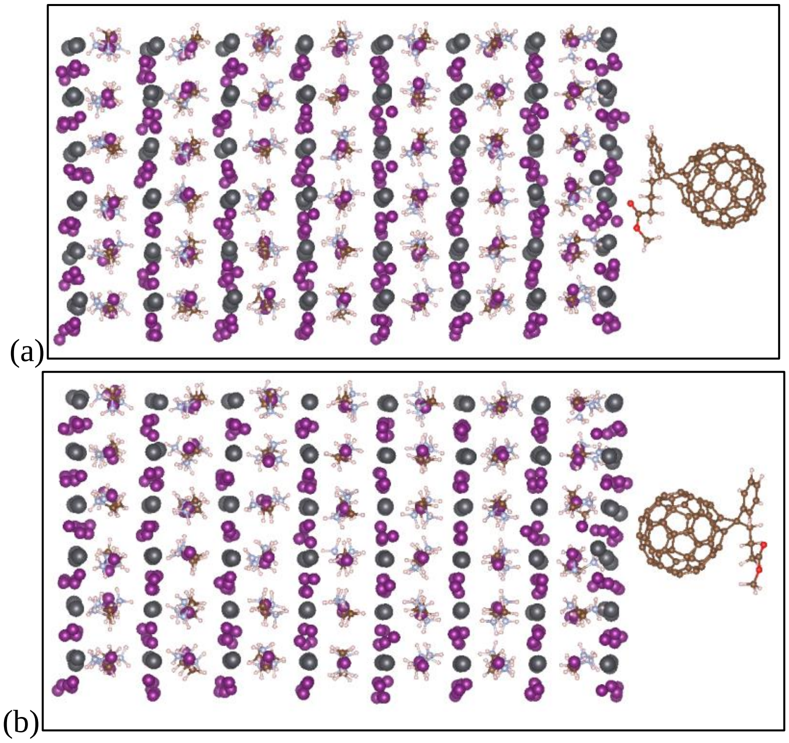

Supplementary Figure 5 | Configurations studied. PCBM attached to perovskite in **(a)** O-facing configuration and **(b)** C_{60}-facing configuration.

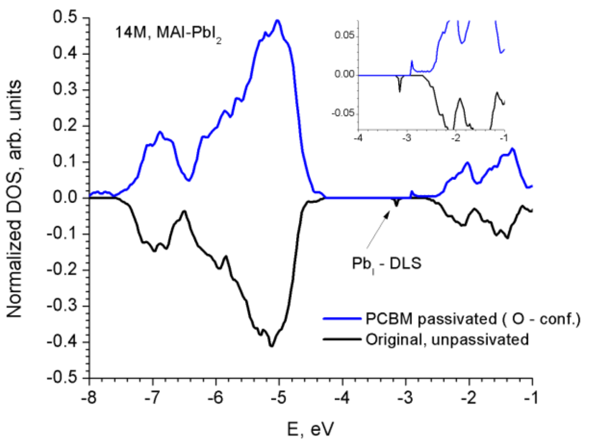

(a)

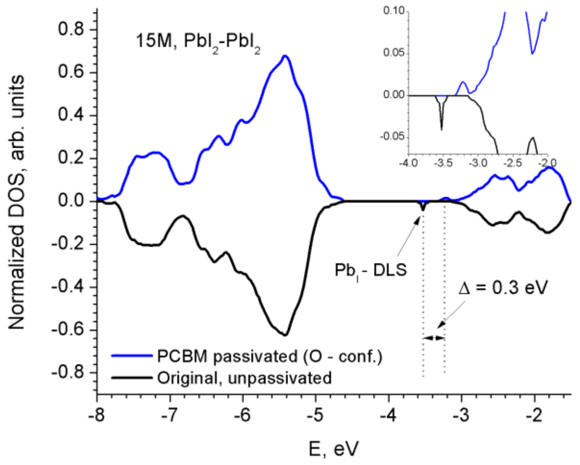

(b)

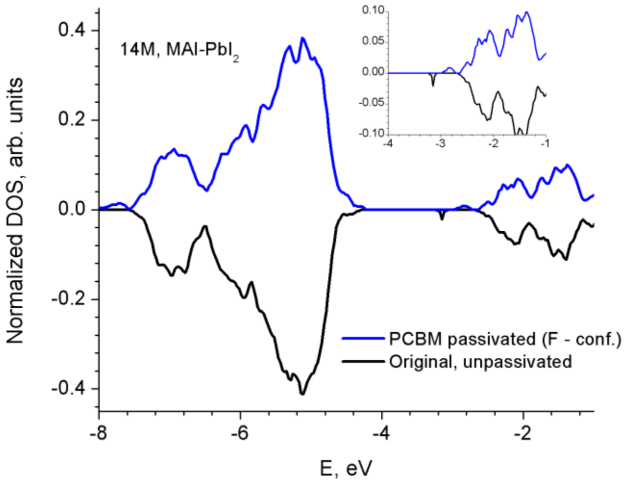

(c)

Supplementary Figure 6 | Density of states (DOS) of defective surface and PCBM + defective surface. DOS have been aligned using deep lying Pb levels.

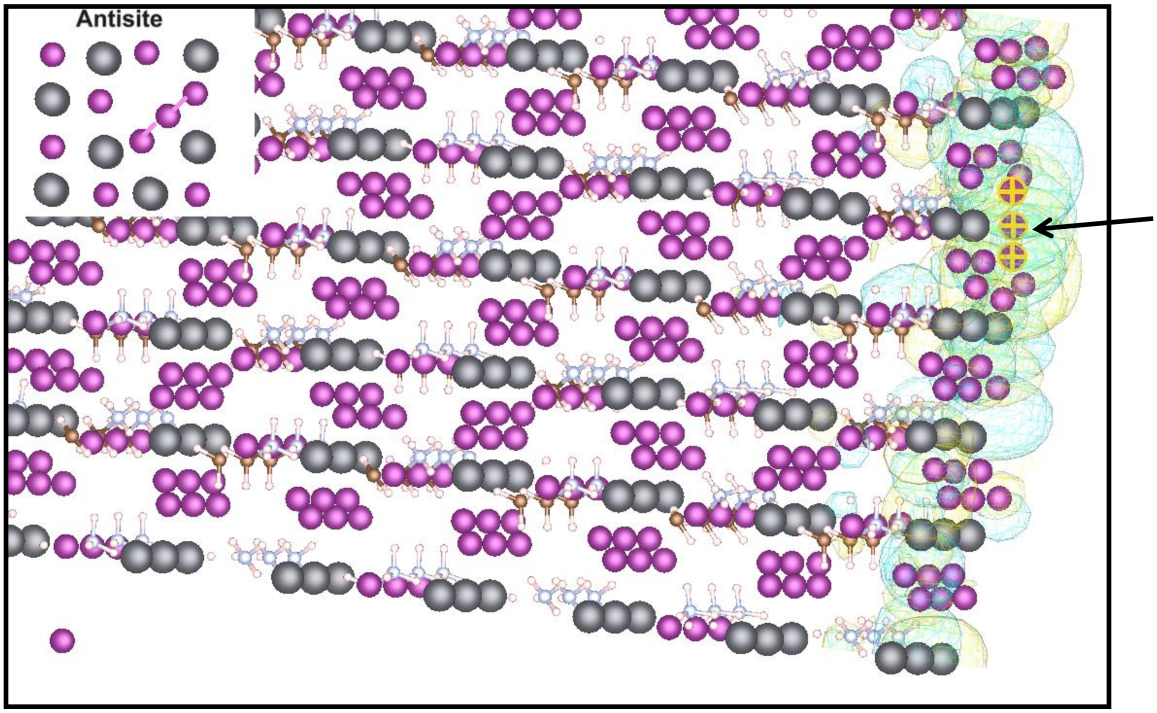

Supplementary Figure 7 | Pb-I antisite defect on perovskite grain surface. The lead (grey) atom is substituted by iodine atom (purple), indicated by arrow. The antisite iodine atom shows stronger covalence with adjacent iodine atoms, forming the I_3 trimer. Pb-I antisite defect is the most anticipated deep trap in perovskite.

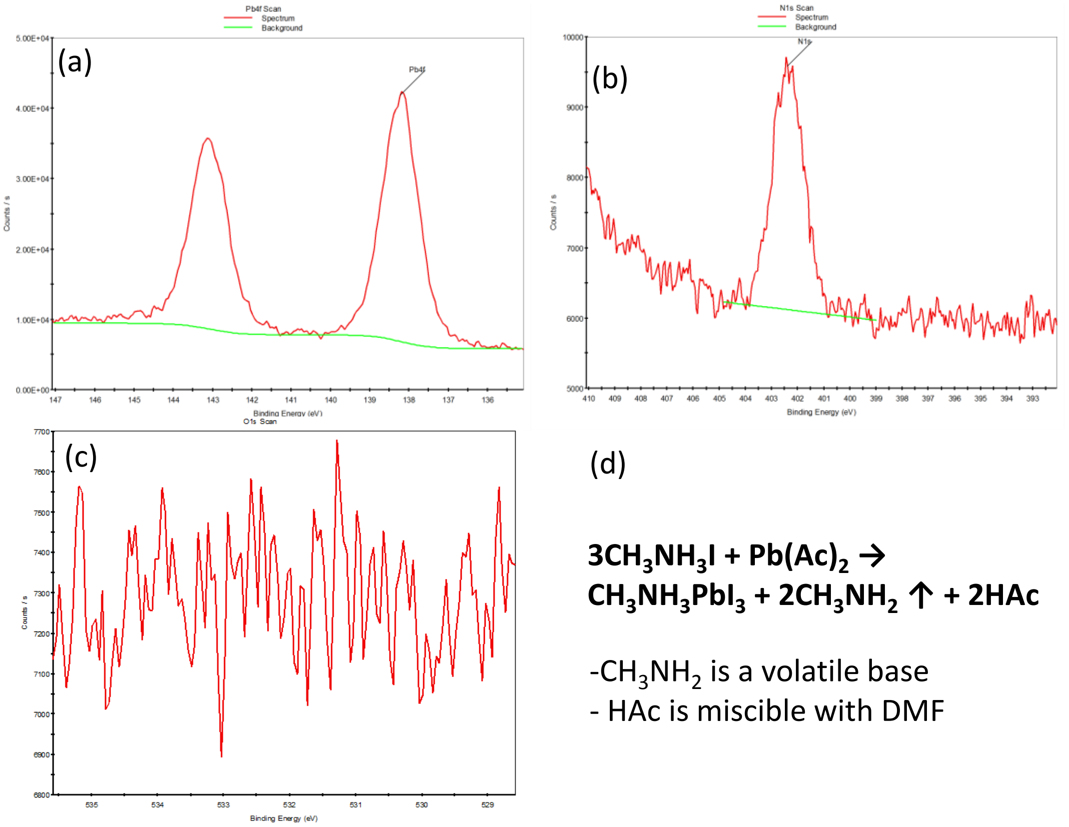

Supplementary Figure 8 | XPS study of one-step $MAPbI_3$ perovskite based on $Pb(Ac)_2$ precursor. **(a)** XPS analysis on lead (Pb) in perovskite film and **(b)** nitrogen (N) element. The area under curves corresponds to 1:1 Pb:N stoichiometry; **(c)** XPS analysis on oxygen (O) element to show no acetate ion left in perovskite film. **(d)** Proposed reaction routine for the pure perovskite formed from $Pb(Ac)_2$ and MAI with molar ratio 1:3.

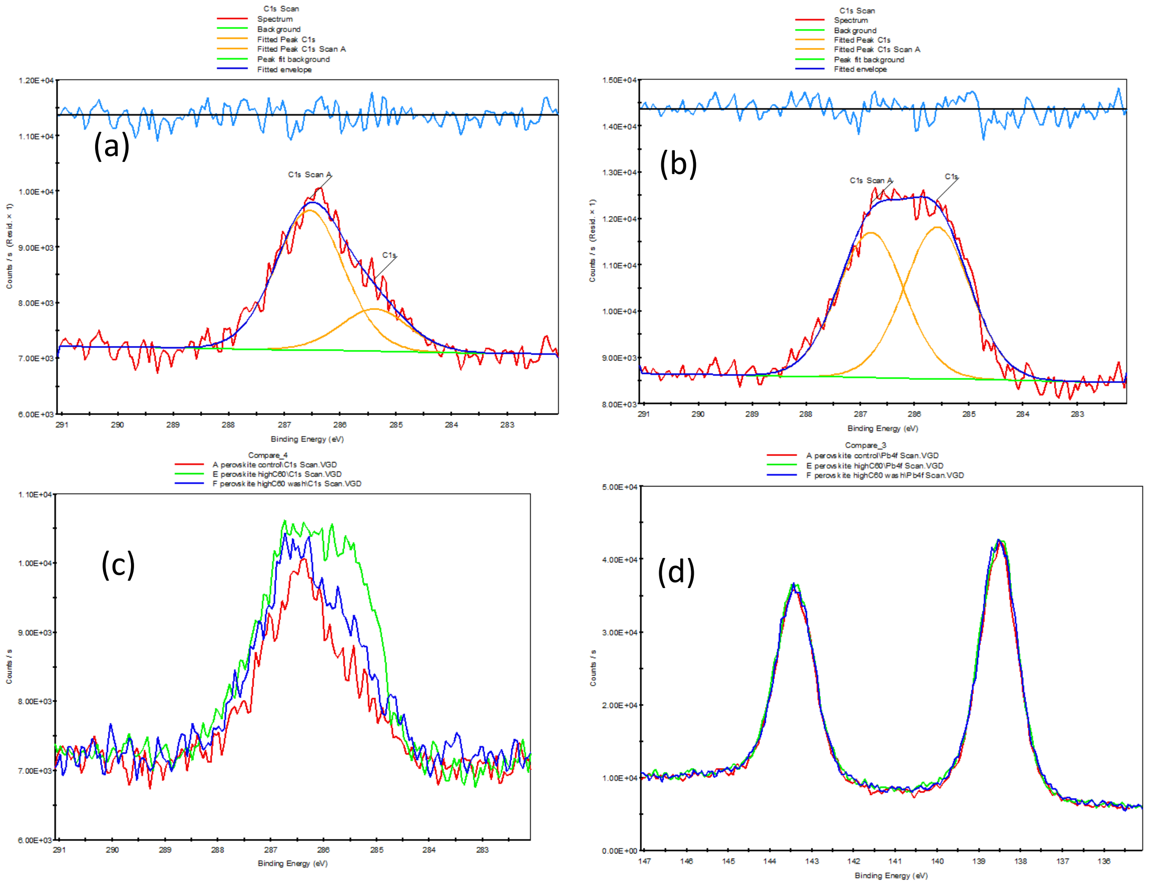

Supplementary Figure 9 | XPS study on Perovskite-PCBM hybrid film during device fabrication. **(a)** Carbon elemental analysis on control film (pure perovskite). Methylammonium peak is at 286.6 eV and some adventitious carbon at 285.5 eV. **(b)** Perovskite-PCBM hybrid film shows the presence of extra carbon at 285.4 eV corresponding to added C_{60}; **(c)** Comparison of surface carbon amount between perovskite film (red), perovskite-PCBM hybrid film (green) and perovskite-PCBM hybrid film post-washed by chlorobenzene solvent (blue); Partial loss of C_{60} on surface is observed due to solvent wash. **(d)** Lead (Pb) element comparison between perovskite film (red), perovskite-PCBM hybrid film (green) and perovskite-PCBM hybrid film post-washed by chlorobenzene solvent (blue).

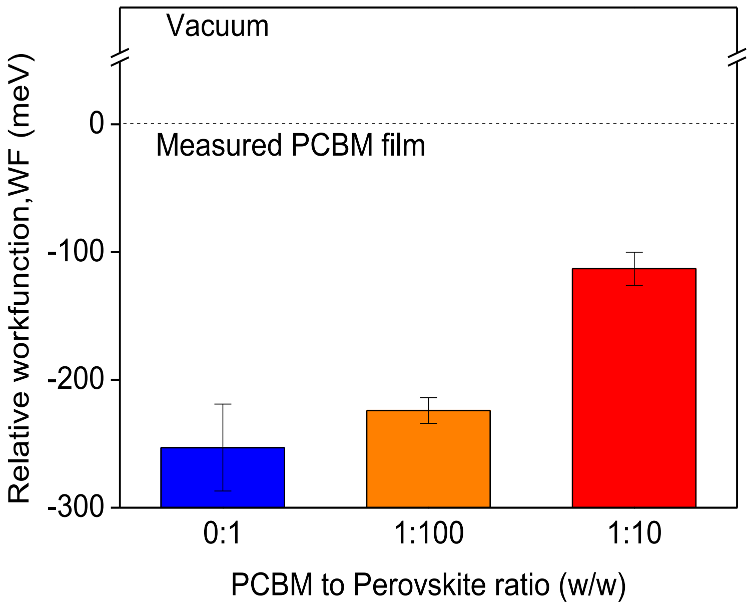

Supplementary Figure 10 | Workfunction of hybrid films. Kelvin probe is used to study the relative work function (WF) of hybrid films with different PCBM ratio. The WF of the hybrid films is pinned between pure perovskite film and pure PCBM film (dashed line). Upon increasing the ratio of PCBM, the WF progressively approaches the value for pure PCBM, indicating a homogenous mixture of the two components.

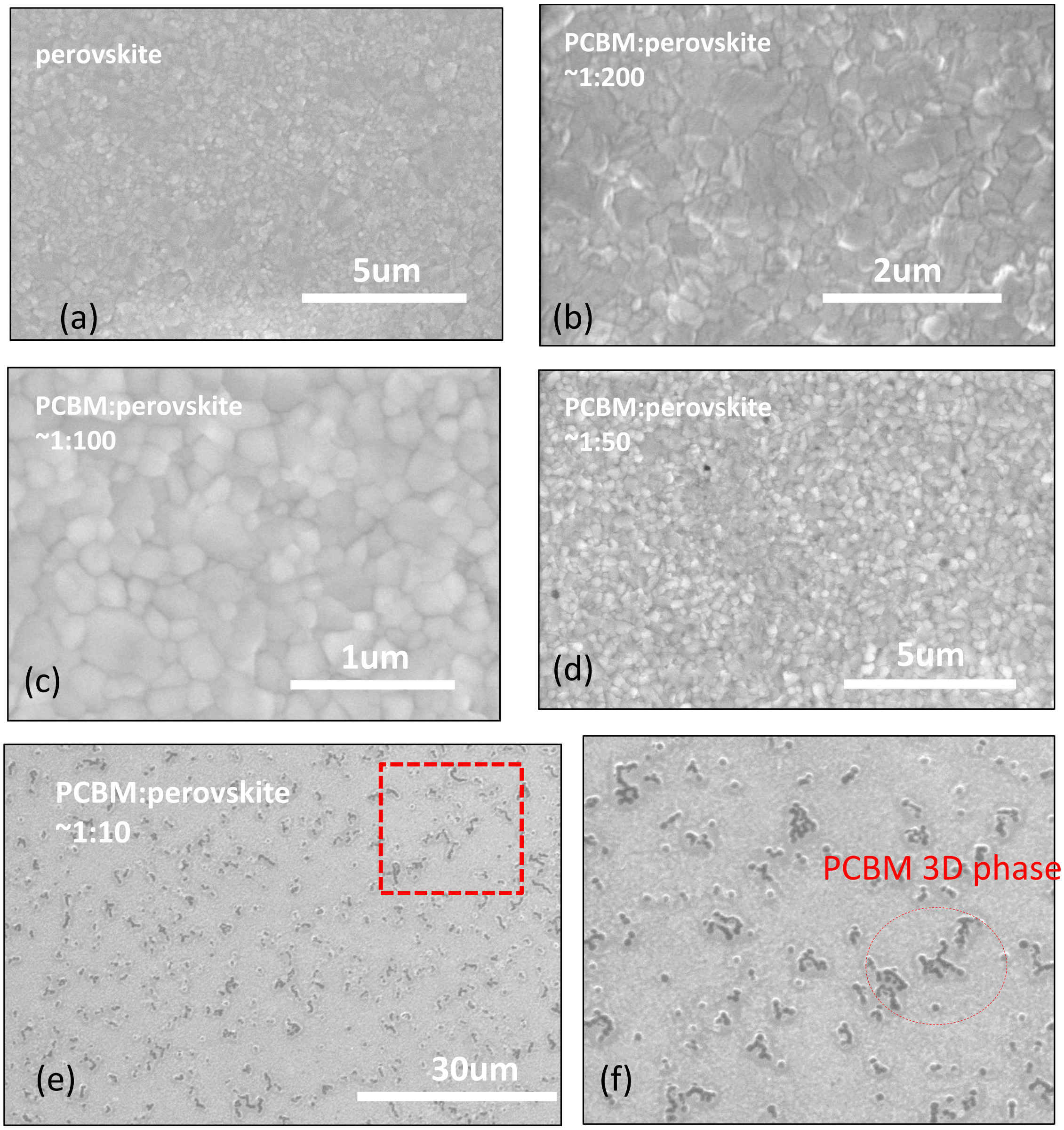

Supplementary Figure 11 | SEM top view. The PCBM phase separation at perovskite grain boundaries becomes visible when PCBM-perovskite hybrid ratio is increased. **(a)** control, perovskite only film; (**b**, **c**, **d, and e**) hybrid films with PCBM ratio progressively increased. (**f**) zoomed in view of a region where PCBM phase emerges at grain boundaries and distributes throughout the film.

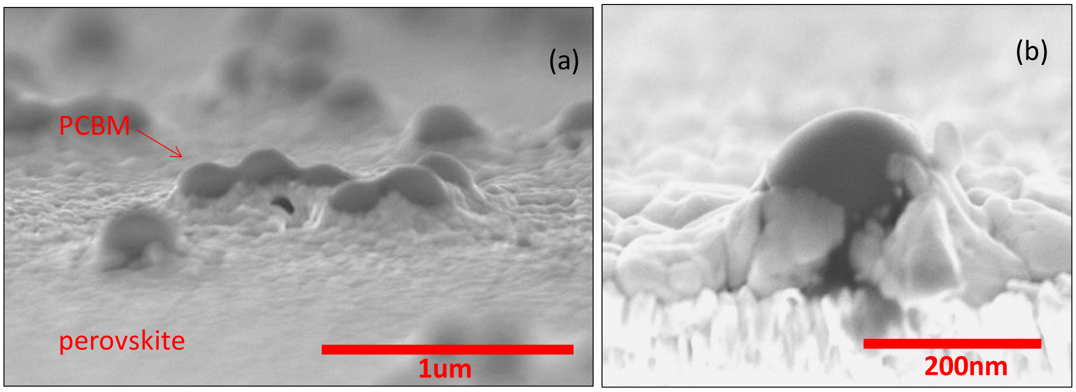

Supplementary Figure 12 | SEM cross-section. 3D phase separation between PCBM and perovskite. **(a)** A mountain-shaped phase separation of PCBM emerges at grain boundaries of perovskite. **(b)** PCBM aggregation throughout the perovskite layer from bottom to top confirms a 3D phase separation.

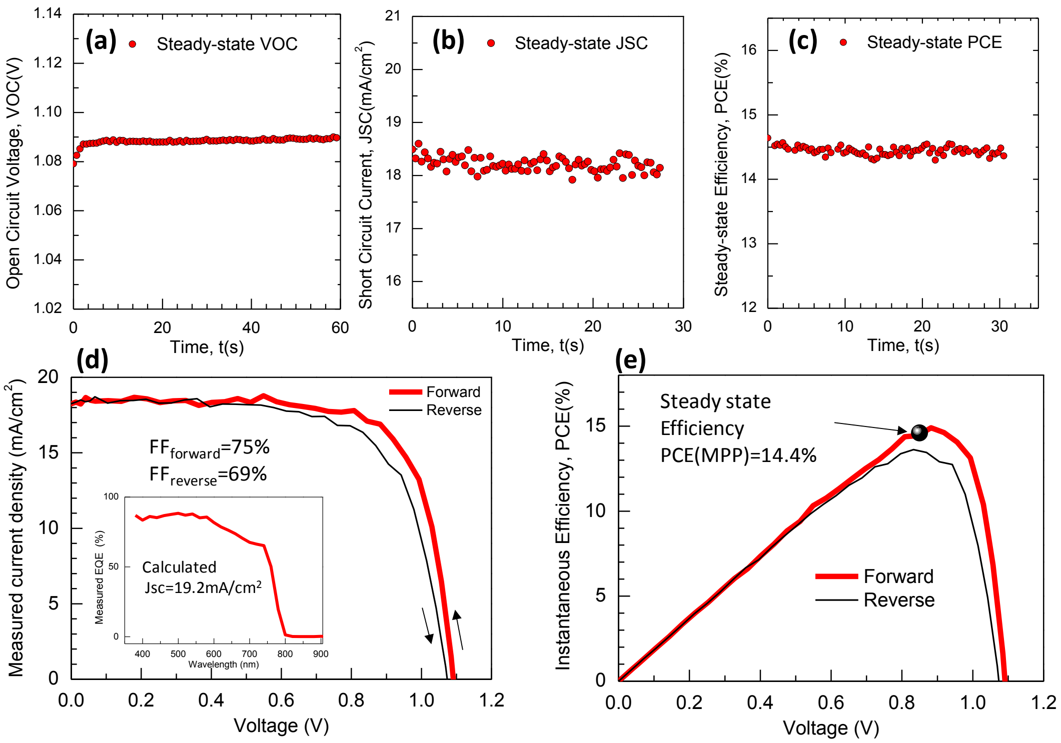

Supplementary Figure 13 | Steady-state photovoltaic performance of champion thick perovskite-PCBM hybrid device. **(a)** The steady state open circuit voltage, V_{OC}, **(b)** steady state short circuit current density, J_{SC}, and **(c)** the steady state power conversion efficiency, PCE, at maximium power point (MPP); (d) The J-V scan of the champion device with very low hysteresis.The inset of **(d)** shows the EQE spectrum of the device. The current density predicted from EQE is 19.2 mA cm^{-2}, consistent with the steady state current density measured in (b); **(e)** The PCE derived from J-V curve. The black point indicates the steady state PCE shown in (**c**). The steady-state MPP is consistent with the forward J-V curve, which indicates the stability of hybrid film.The steady state power conversion efficiency is 14.4%. .

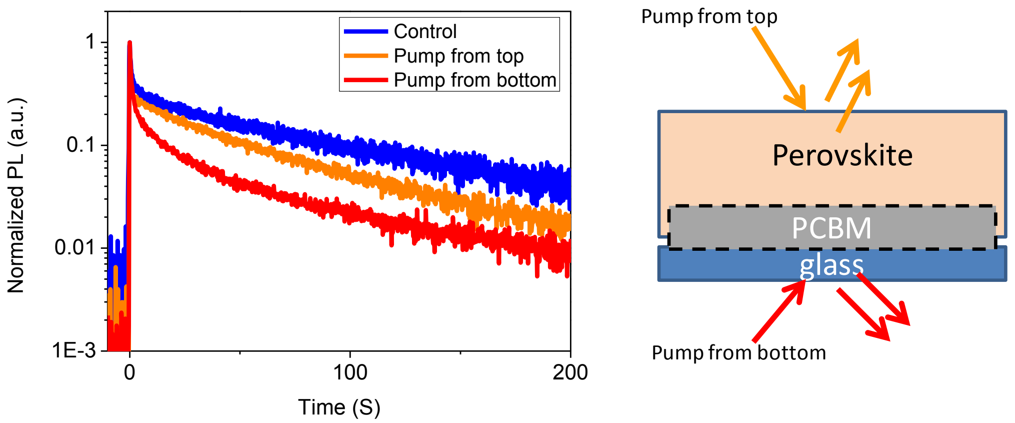

Supplementary Figure 14 | PL decay of PCBM:perovskie “bilayer” film (PCBM at the bottom interface of perovskite), pumped from top and bottom. “Control” is a perovskite-only film.

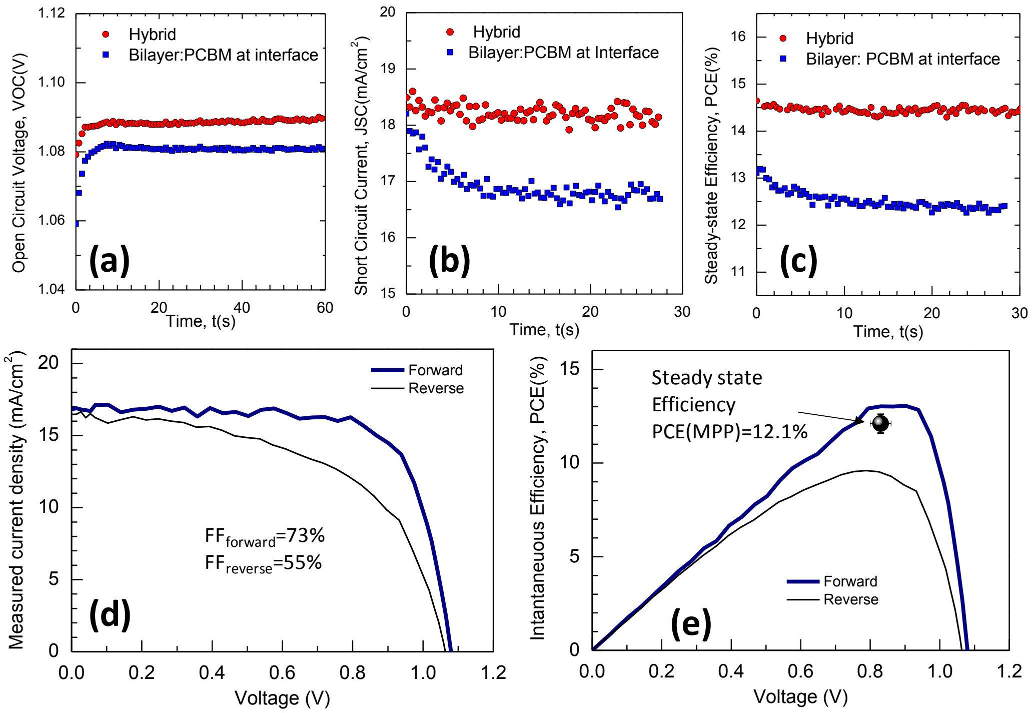

Supplementary Figure 15 | Steady-state photovoltaic performance of a thick “bilayer” device (blue), compared with “Hybrid device” (red). **(a)** The steady state open circuit voltage, V_{OC}, **(b)** steady state short circuit current density, J_{SC}, and **(c)** the steady state power conversion efficiency, PCE, at maximium power point (MPP); **(d)** The J-V scan with large hysteresis in bilayer devices; **(e)** MPP is between two J-V curves, which indicates significant hysteresis and current loss in bilayer devices.

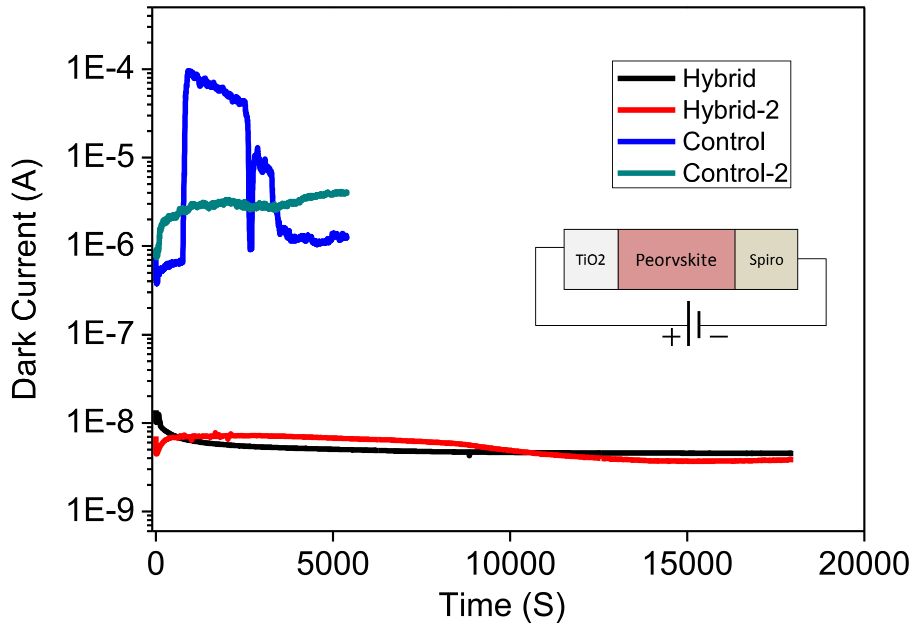

Supplementary Figure 16 | Long-term steady-state dark current measurement of hybrid devices (red and black) and control devices (blue and cyan) under reverse bias -0.5 V. Perovskite-PCBM hybrid devices showed almost 2 orders of magnitude lower dark current and no breakdown during the course of the measurement. Bias is applied continuously and dark current is sampled every 1 second.

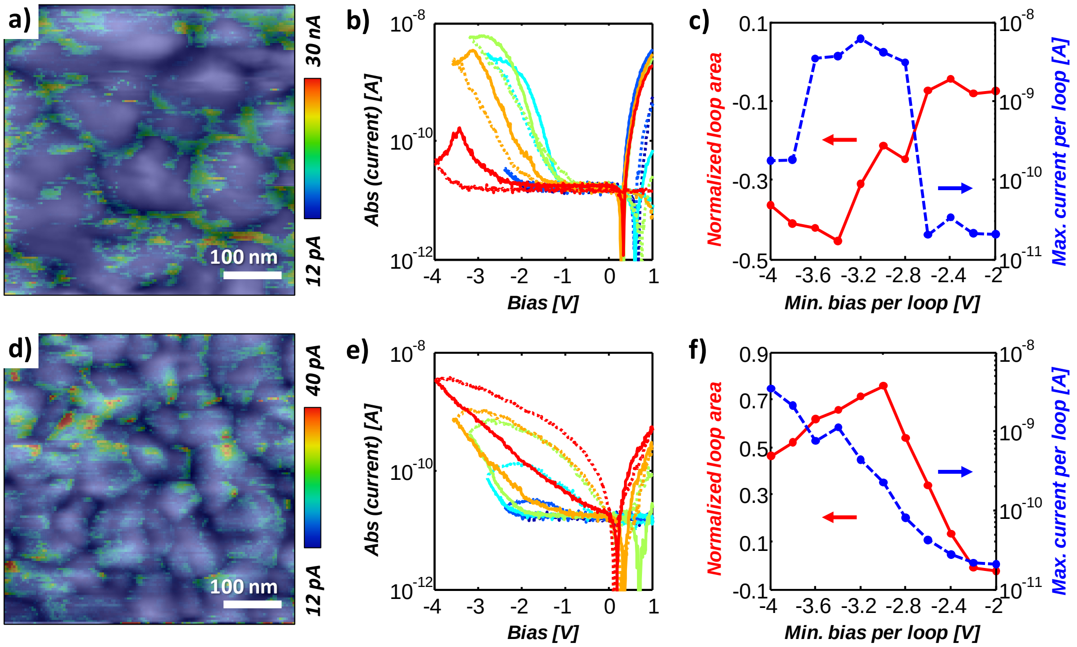

Supplementary Figure 17 | Perovskite **a)**, **b)**, **c)** with and **d)**, **e)**, **f)** without PCBM. **a)** and **d)** Gray-scaled contact-mode AFM (background) with overlaid color-scaled conductive AFM images (sample bias voltage: 1 V). **b)** and **e)** Sequential I-V curves (solid line: forward sweep, dashed line: reverse sweep). Final voltage in each sweep is sequentially lowered down to −2, −2.4, −2.8, −3.2, −3.6, and −4 V (navy to red) maintaining a fixed initial voltage at 1 V, obtained near grain boundary areas of both samples. **c)** and **f)** Normalized loop area versus minimum bias voltage of each loop in **b**) and **c)**, respectively. **a)** and **d**) show conductivity at grain boundary areas is higher than that at grain center areas in both samples. The perovskite sample treated with PCBM has much higher conductivity near grain boundary areas at positive sample bias voltages, which points to improved electron extraction from PCBM at grain boundaries. Significant hysteresis is consistently observed in perovskite control sample (**e**).

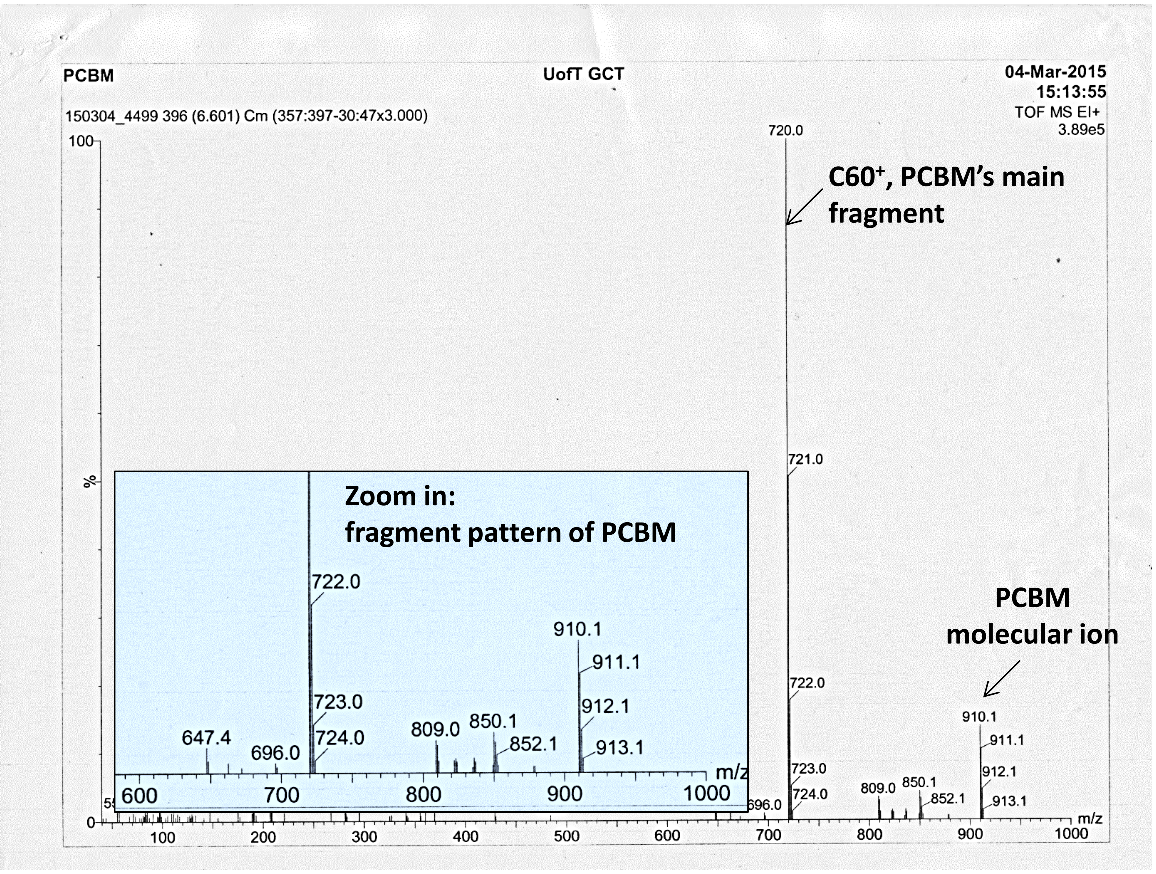

Supplementary Figure 18 | Time-of-flight mass spectrometry (TOF-MS) of solid films of hybrid device by EI (Electron Ionization). The solid film is the perovskite-PCBM hybrid film capped with Spiro-OMeTAD atop. The molecular ion of PCBM is clearly visible at m/z = 910.1. The fragment pattern of PCBM is shown in the zoom-in figure (inset). The main fragment of PCBM is shown at the base peak m/z = 720, which is C_{60}+. This result confirms that PCBM remains in the perovskite-PCBM hybrid film following the casting of Spiro-OMeTAD on top.

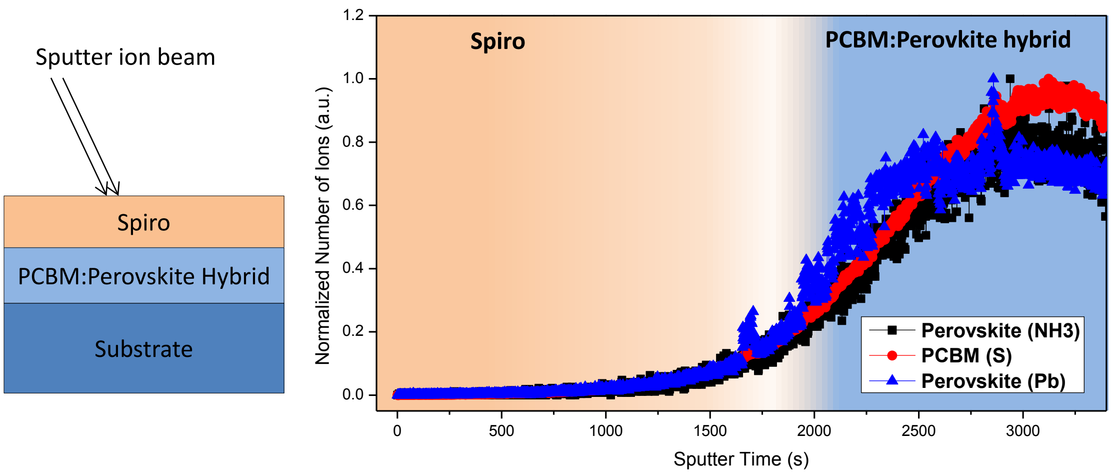

Supplementary Figure 19 | Secondary Ion Mass Spectrometry (SIMS) depth profile of perovskite-PCBM hybrid film with Spiro-OMeTAD layer on top. The sputtering begins at the air/Spiro-OMeTAD interface and goes down to the Spiro-OMeTAD/Hybrid film interface. PCBM is tracked by the S^+ ions from a sulfur-stained [60]ThPCBM which is analogous to PCBM used in devices; $CH_3NH_3PbI_3$ perovskite is tracked by Pb^+ and NH_3^+ ions; The distribution profile of PCBM in the film follows the profile of perovskite, indicating no major changes in PCBM concentration near the top of the film.

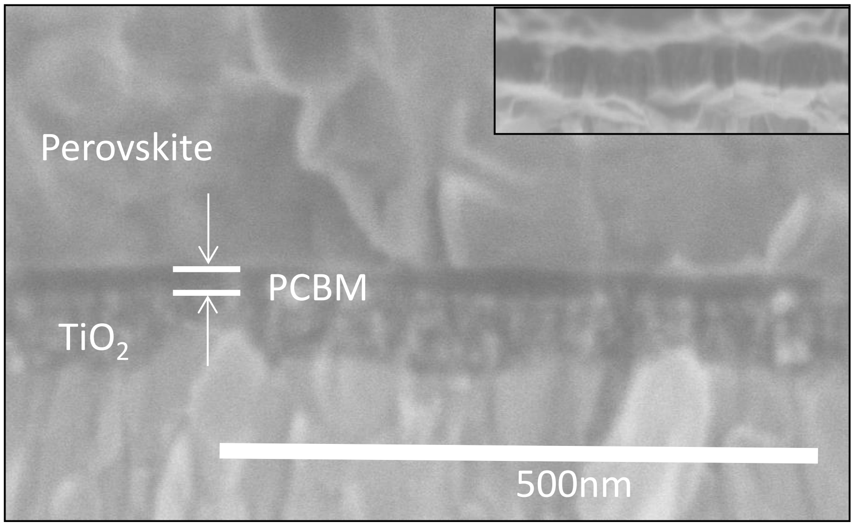

Supplementary Figure 20 | Interface of TiO2/PCBM/Perovskite "bilayer" device. A thin layer of PCBM is formed between the planar perovskite film and TiO2 substrate; and (Inset) zoom-in on a PCBM layer.

Supplementary Table 1 | Binding energies of PCBM to the defect-free perovskite surface in vacuum.

$E_{ads.}^{PCBM}$, eV	Configuration	Slab, N of monol., PbI_2 side
0	O-facing	15 (PbI_2-PbI_2)
-0.37	O-facing	14 (MAI-PbI_2)
0	C_{60}-facing	15 (PbI_2-PbI_2)
-0.12	C_{60}-facing	14 (MAI-PbI_2)

Supplementary Table 2 | Antisite defect formation energies on (100) perovskite PbI_2-terminated surface.

H_f, eV I-rich conditions	H_f, eV I-poor conditions	Site number	Slab, N of monol., PbI_2 side
0.27	3.65	1	14
0.11	3.49	2	14
0.03	3.41	1	15
0.22	3.60	2	15
0.05	3.43	3	15

Supplementary Table 3 | Binding energies of PCBM to the defective perovskite surface in vacuum.

$E_{ads.}^{PCBM}$, eV	Configuration	Slab, N of monol., PbI_2 side
-0.3	O-facing	15 (PbI_2-PbI_2)
-0.7	O-facing	14 (MAI-PbI_2)
-0.1	C_{60}-facing	15
-0.3	C_{60}-facing	14

Supplementary Table 4 | Grain size (unit: Å) of perovskite film estimated from XRD peak area

Film	Bragg peak			
	002	004	006	400
Control-1	525.968	580.12	555.55	630.316
Control-2	417.707	513.48	556.47	562.807
Hybrid-1	608.635	512.661	618.333	630.316
Hybrid-2	608.635	580.01	585.05	630.316

Supplementary Note 1 | Density functional theory study of PCBM in-situ passivation effect on perovskite in hybrid solid

Calculations were performed within the Density Functional (DFT) formalism using the Perdew-Burke-Ernzerhof (PBE)[1] GGA exchange correlation functional. All calculations were performed utilizing the CP2K[2] package within Gaussian-augmented plane waves (GAPW) dual basis set using the molecularly optimized MOLOPT[3] double ζ -valence polarized (mDZVP) basis set implemented in CP2K code which has very small Basis Set Superposition errors (BSSE) in gas and condensed phases[4-7]. The grid cutoff was 300 Ry, which is suitable for the Goedecker-Teter-Hutter pseudopotentials[8]. Spin polarized (LSDA) and spin-unpolarized caculations (LDA) were performed in the case of the odd and even number of electrons, respectively. The structural minimization was performed with the help of the Broyden-Fletcher-Goldfarb-Shanno algorithm[9] (BFGS).

We have modelled the passivation of the perovskite surface with PCBM ligand. Surface slabs were modelled as (001) terminated slabs of tetragonal structure with 14 and 15 interchanging monolayers i.e. stoichiometric asymmetric slab (MAI-PbI_2) and off-stoichiometric symmetric slabs (PbI_2-PbI_2 or MAI-MAI termination). In light of recently proposed ferroelectricity of the perovskite [10] we have studied polar stoichiometric slabs i.e. even - numbered slabs (in our case 14 monolayers). It was found previously[11] that unreconstructed (001) tetragonal termination possesses low surface energy and as a result represents the most probable surface termination. 100 Å of vacuum was added on top of the slab surface (with and without PCBM attached to the surface for consistency). Dipole-slab correction was used to remove artificial dipole-dipole interaction across periodic images in vacuum as implemented in the CP2K code of version 2.5. A 3x3 (26.88 Å x 26.88 Å) periodicity was used in the xy-plane. The basis set superposition error (BSSE)[4, 12]in PCBM binding energy was estimated using the counterpoise correction method[13] to be ~3 meV and was subsequently neglected. To create dipole-free slab in case of symmetric terminations we have performed the Born-Openheimer molecular dynamics in NVT ensemble with 0.5 fs time step over the 10 ps to allow methylammonium rotational degrees of freedom to smoothen out and chose the configurations with net-zero dipole along normal to the perovskite surface (001), subsequently relaxed (Supplementary Figure 3). Similarly we have chosen the structures with mean dipole moment in the case of the even-number asymmetric slabs. Such approach provides configurations that are ensemble representative at 300 K. No band bending was observed in the case of 14M case (however at larger thickness band bending becomes apparent), which could be explained by the topmost mobile MAI layer partially compensating the built-in electric field due to non-zero dipole moment as can be seen from Supplementary Figure 3. Supplementary Figure 4 shows the density of states (DOS) of symmetric off-stoichiometric slabs (15 monolayers) where one can see no in-gap states.

To demonstrate the passivation effect caused by PCBM, we have concentrated on PbI_2-terminated surface as it was shown to posses small surface energy[11] and therefore be very stable. PCBM was attached in two configuration as depicted in Supplementary Figure 5.

Adsorption energies were calculated using the Supplementary Equation 1:

$$E_{ads.}^{PCBM} = E(PCBM + Slab)_{relaxed} - E(Slab)_{relaxed} - E(PCBM)_{relaxed}. \quad (1)$$

The binding energies are given in the Supplementary Table 1 and Supplementary Table 3. Negative values indicate binding.

One can see that in the case of symmetrically off-stoichiometric slab (PbI_2 - PbI_2) PCBM easily desorbs whereas in the case of stoichiometric polar slab, binding energies correspond to weak physisorption.

To understand electronic properties further, we took the most anticipated defect [14] in bulk, namely Pb_I antisite (Pb - atom is being substituted by I atom) and performed defect formation energies calculations (H_f) on the surface (Supplementary Equation 2) to verify that it maintains the low formation energy and forms a trap state in the gap as observed in the bulk.

$$H_f = E^{DFT}(def.slab) - E^{DFT}(slab) - \mu_{Pb}^{f.c.c} + \frac{1}{2}\mu_{I_2^{bulk}} \quad (2)$$

where $E^{DFT}(def.slab)$ - is the energy of slab with defects, $E^{DFT}(slab)$ - is the energy of the defect-free slab, and $\mu_{Pb}^{f.c.c}$, $\frac{1}{2}\mu_{I_2^{bulk}}$ - are the chemical potentials of the Pb and I atoms in their stable states at standard conditions. The defect formation energies at various surface sites are listed in the Supplementary Table 2.

It can be seen that the defect formation energies are not affected by polarity and have very small values in the case of I-rich conditions; thus, indicating high probability of existence on the surface. Carefully chosen lead precursor such as lead acetate $Pb(Ac)_2$ can aid in the removing the traps up to certain degree [7]but not completely.

The DOS in case of surface Pb_I trap alone and with PCBM attached to the defect is shown at Fig. S6. One can see that traps become shallower, consistent with the observation of higher V_{oc} upon mixing with PCBM. The trap level shifts due to the charge transfer between PCBM and surface. Mulliken and Bader charge analysis consistently gave values of ~0.2 electrons being transferred to the surface from the PCBM.

By looking at the PCBM binding energies to the defective surface in Supplementary Table 3, one can see that it went from weak physisorption to a stronger binding. It is seen on the Fig. S6 that both configurations (O-facing and C_{60}(F)-facing) can passivate the Pb_I defect; however, the fullerene-facing (F-configuration) better passivates the polar (14 monolayers) slab. This points toward the nature of interaction being the electrostatic, and halogen-bond-like type (D—O - I — A)[15].

Binding energies of the PCBM attached to the defective slabs can be rewritten as differences in defect formation energies of the Pb_{PCBM_I} and Pb_I with defect formation energy of the Pb_{PCBM_I} being lower by the amount of binding energy, thus making it more preferable to form Pb_{PCBM_I} rather than Pb_I defect. Supplementary Figure 7 shows localization pattern of the Pb_I defect trap state.

Supplementary Note 2 | Mass Spectrometry of PCBM-perovskite hybrid device

From this dataset, we verify the presence and distribution of PCBM in blended film after spiro-OMeTAD is spin cast on top. We proposed the possible reasons why PCBM remains in the film: a) the very brief contact time (no soaking time) between hybrid film and spiro-OMeTAD solution, aided by the fast spin-coating speed (4000-5000 rpm); b) the good coupling between PCBM and perovskite and c) the protection of re-crystallized PCBM by the dense, compact grain of perovskite films after annealing.

Method of Time-of-flight Mass Spectrometry (TOF-MS, Waters GCT Premier): 1) “Raw sample solution”: The device-analogous solid film (Perovskite:PCBM hybrid layer + Spiro-OMeTAD atop) was fully dissolved by DMF and chlorobenzene mixture solvent to obtain the “raw sample solution”. 2) Extraction of PCBM from raw sample solution: the sample solution was poured into water, and extracted with chloroform, and then washed with copious of water three times to remove the Perovskite salts. The separated organic phase was dried with MgSO4, getting a “resultant solution” which might contain PCBM. The resultant solution was Rotary-evaporated and a brown solid was obtained. The brown solid was re-dissolved into $CHCl_3$. 3) Mass spectrometry: The $CHCl_3$ solution of the extracted solid was submitted to measure its time-of-flight mass spectrometry by EI (Electron ionization, Waters GCT Premier). The solution was dried before loading the sample.The result is shown in Supplementary Figure 18.

Supplementary Methods

XRD study

Perovskite active layer thickness was estimated as 150 nm via SEM cross-section. The substrate consisted of a TiO_2 compact layer on FTO coated glass. Grain sizes (unit: Å, Supplementary Table 4) were estimated from area of 002, 004, 006 and 400 Bragg peaks of the XRD spectra.

XPS study on stochiometery

X-ray photoelectron spectroscopy (XPS) is carried out using a Thermo Scientific K-Alpha spectrometer. Core level spectra of Pb-4f, I-3d, O-1s, N-1s and C-1s with a pass energy of 75 eV. The elemental composition was calculated based on integrated counts of respective peaks. The curves were fitted using Gaussian functions with 1.5 eV FWHM. For comparison of different samples, all spectra were normalized to Pb signal. The results are shown in Supplementary Figure 8 and 9.

Supplementary references

(1) Perdew, J. P., Burke, K., Ernzerhof, M. Generalized Gradient Approximation Made Simple. *Phys. Rev. Lett.* **77**, 3865-3868 (1996).

(2) VandeVondele, J., Krack, M., Mohamed, F., Parrinello, M., Chassaing, T., Hutter, J. QUICKSTEP: Fast and accurate density functional calculations using a mixed Gaussian and plane waves approach. *Comp. Phys. Comm.* **167**(2), 103-128 (2005)

(3) VandeVondele, J., Hutter, J. Gaussian basis sets for accurate calculations on molecular systems in gas and condensed phases. *J. Chem. Phys.* **127**, 114105 (2007).

(4) Leitsmann, R., Bohm, O., Planitz, P., Radehaus, C., Schaller, M., Schreiber, M. Adsorption mechanisms of fluorocarbon polymers at ultra low-k surfaces. *Surf. Sci.* **604**, 1808 - 1812 (2010).

(5) Takaluoma, T. T. , Laasonen, K., Laitinen, R. S. Molecular Dynamics Simulation of the Solid-State Topochemical Polymerization of S_2N_2. *Inorg. Chem.* **52**, 4648-4657 (2013).

(6) Bork, N., Loukonen, V., Vehkamki, H., Reactions and Reaction Rate of Atmospheric SO_2 and O-3(-) $(H_2O)_{(n)}$ Collisions via Molecular Dynamics Simulations. *J. Phys. Chem. A* **117**, 3143-3148 (2013).

(7) Smecca, E., Motta, A., Fragal, M. E., Aleeva, Y., Condorelli, G. G. Spectroscopic and Theoretical Study of the Grafting Modes of Phosphonic Acids on ZnO Nanorods. *J. Phys. Chem. C* **117**(10), 5364-5372 (2013).

(8) Hartwigsen, C., Goedecker, S., Hutter, J. Relativistic separable dual-space Gaussian pseudopotentials from H to Rn. *Phys. Rev. B* **58**, 3641-3662 (1998).

(9) Press, W. H.; Teukolsky, S. A., Vetterling, W. T., Flannery, B. P. Numerical Recipes 3rd Edition: The Art of Scientic Computing, 3rd ed.; Cambridge University Press: New York, NY, USA, 2007.

(10) Frost, J. M., Butler, K. T., Walsh, A., Molecular ferroelectric contributions to anomalous hysteresis in hybrid perovskite solar cells. *APL Materials* **2**(8), 081506 (2014).

(11) Haruyama, J., Sodeyama, K., Han, L., Tateyama, Y. Termination Dependence of Tetragonal $CH_3NH_3PbI_3$ Surfaces for Perovskite Solar Cells. *Phys. Chem. Lett.* **5**(16), 2903-2909 (2014).

(12) Jansen, H. B., Ros, P. Non-empirical molecular orbital calculations on the protonation of carbon monoxide. *Chem. Phys. Lett.* **3**, 140-143 (1969).

(13) Boys, S. F., Bernardi, F. The calculation of small molecular interactions by the differences of separate total energies. Some procedures with reduced errors. *Mol. Phys.* **19**, 553-566 (1970).

(14) Buin, A., Pietsch, P., Xu, J., Voznyy, O., Ip, A.H., Comin, R., Sargent, E.H. Materials Processing Routes to Trap-Free Halide Perovskites. *Nano Lett.* **14**(11), 6281-6286 (2014).

(15) Abate, A., Saliba, M., Hollman, D. J., Stranks, S. D., Wojciechowski, K., Avolio, R., Grancini, G., Petrozza, A., Snaith, H. J. Supramolecular halogen bond passivation of organic–inorganic halide perovskite solar cells. *Nano Lett.* **14**(6), 3247-3254 (2014).
